# Core outcome set measurement for future clinical trials in acute myeloid leukemia: the HARMONY study protocol using a multi-stakeholder consensus-based Delphi process and a final consensus meeting

**DOI:** 10.1186/s13063-020-04384-1

**Published:** 2020-05-27

**Authors:** Katharina M. Lang, Kathryn L. Harrison, Paula R. Williamson, Brian J. P. Huntly, Gert Ossenkoppele, Jan Geissler, Tamàs Bereczky, Jesús M. Hernández-Rivas, Hélène Chevrou-Séverac, Rory Goodbody, Renate Schulze-Rath, Lars Bullinger

**Affiliations:** 1 Charité University Medicine, Chariteplatz 1, Berlin, 10117 Germany; 2 NICE, National Institute for Health and Care Excellence, 10 Spring Gardens, London, SW1A 2BU UK; 3 MRC North West Hub for Trials Methodology Research, University of Liverpool, Brownlow Hill, Liverpool, L69 7ZX UK; 4 University of Cambridge, Trinity Lane, Cambridge, CB2 1TN UK; 5 VU University Medical Center, De Boelelaan 1105, Amsterdam, 1081 HV Netherlands; 6 Patvocates, Am Rothenanger 1b, Riemerling, 85521 Germany; 7 Universidad de Salamanca, Patio de Escuelas, 1, Salamanca, 37008 Spain; 8 Celgene International, Route de Perreux 1, Boudry, CH-2017 Switzerland; 9 AbbVie, 14 Riverwalk, Dublin, D24 XN32 Ireland; 10 Bayer AG, Müllerstraße 178, Berlin, 13353 Germany

**Keywords:** Delphi process, Core outcome set, Acute myeloid leukemia

## Abstract

**Background:**

Acute myeloid leukemia (AML) is the most common acute leukemia in adults and has an unacceptably low cure rate. In recent years, a number of new treatment strategies and compounds were developed for the treatment of AML. There were several randomized controlled clinical trials with the objective to improve patients’ management and patients’ outcome in AML. Unfortunately, these trials are not always directly comparable since they do not measure the same outcomes, and currently there are no core outcome sets that can be used to guide outcome selection and harmonization in this disease area. The HARMONY (Healthcare Alliance for Resourceful Medicine Offensive against Neoplasms in Hematology) Alliance is a public-private European network established in 2017 and currently includes 53 partners and 32 associated members from 22 countries. Amongst many other goals of the HARMONY Alliance, Work Package 2 focuses on defining outcomes that are relevant to each hematological malignancy. Accordingly, this pilot study will be performed to define a core outcome set in AML.

**Methods:**

The pilot study will use a three-round Delphi survey and a final consensus meeting to define a core outcome set. Participants will be recruited from different stakeholder groups, including patients, clinicians, regulators and members of the European Federation of Pharmaceutical Industries and Associations. At the pre-Delphi stage, a literature research was conducted followed by several semi-structured interviews of clinical public and private key opinion leaders. Subsequently, the preliminary outcome list was discussed in several multi-stakeholder face-to-face meetings. The Delphi survey will reduce the preliminary outcome list to essential core outcomes. After completion of the last Delphi round, a final face-to-face meeting is planned to achieve consensus about the core outcome set in AML.

**Discussion:**

As part of the HARMONY Alliance, the pilot Delphi aims to define a core outcome set in AML on the basis of a multi-stakeholder consensus. Such a core outcome set will help to allow consistent comparison of future clinical trials and real-world evidence research and ensures that appropriate outcomes valued by a range of stakeholders are measured within future trials.

## Introduction

Acute myeloid leukemia (AML) is the most common acute leukemia in adults. Clonal expansion of undifferentiated myeloid precursor cells causes impaired hematopoiesis and bone marrow failure. For decades, the basis of AML treatment has remained virtually unchanged. However, over the last few years, driver mutations have been identified and molecular subgroups defined, resulting in an improved prognostic stratification and updated European LeukemiaNET guidelines [1, 2].

Although scientific and technical advances have resulted in a number of new treatment strategies in recent years, AML still poses a challenge to curative approaches: cure rates remain poor compared with those of other hematological malignancies. Currently, several innovative compounds are being investigated in randomized controlled clinical trials with the objective to improve both patients’ outcome and patients’ management in AML. However, the ability to compare these clinical trials is limited by differences in their measured outcomes. This lack of standardization relates to the current lack of a core outcome set (COS) that can be used to guide outcome selection and harmonization in AML in current and future trials.

The HARMONY (Healthcare Alliance for Resourceful Medicine Offensive against Neoplasms in Hematology) Alliance is a public-private European network established in 2017 and currently includes 53 partners, *inter alia* six cancer patient umbrella organizations, and 32 associated members from 22 countries. One of HARMONY’s goal is to use big data to improve understanding and treatment of hematological malignancies. In order to achieve this aim, HARMONY is structured into eight work packages, of which Work Package 2 (WP 2) is focused on defining outcomes that are relevant to each hematological malignancy. In the future, WP 2 aims to define a common COS valid for all hematological malignancy. Accordingly, this pilot study will be performed to define a COS in AML.

A COS is a minimum set of outcomes developed by consensus, usually using a multi-stakeholder consensus-based Delphi process. The COS is a reference point and provides the minimum outcome set that should be collected in further clinical trials on a given condition. Use of a COS improves comparability of clinical trials and consistency of reporting, reduces selective reporting bias and ensures that appropriate outcomes valuable by a range of stakeholders are measured. Furthermore, a COS can be used in other clinical settings or type of real-world evidence research and can be incorporated into clinical guidelines and improve clinical practice in this way.

Involvement of different stakeholder groups is critical to ensure that the defined COS has broad relevance. Key stakeholders will provide their expert feedback and will be recruited on the basis of their experiences relevant to AML or the project. The different stakeholder groups include health service users, health service practitioners, researchers, drug developers, regulators, patient advocates and patients. All ratings are provided anonymously; so without group interactions, different opinions will be presented more clearly between different stakeholder groups.

### Aims

This project aims to define a COS in AML agreed by consensus of all stakeholder groups to be measured in future clinical trials and observational studies. This pilot study also aims to establish the Delphi method for further disease-specific COS defining studies that are planned for other hematological malignancies in HARMONY. The defined COS will help to improve future clinical study design to improve patient satisfaction and management.

## Methods

**Fig. 1:**
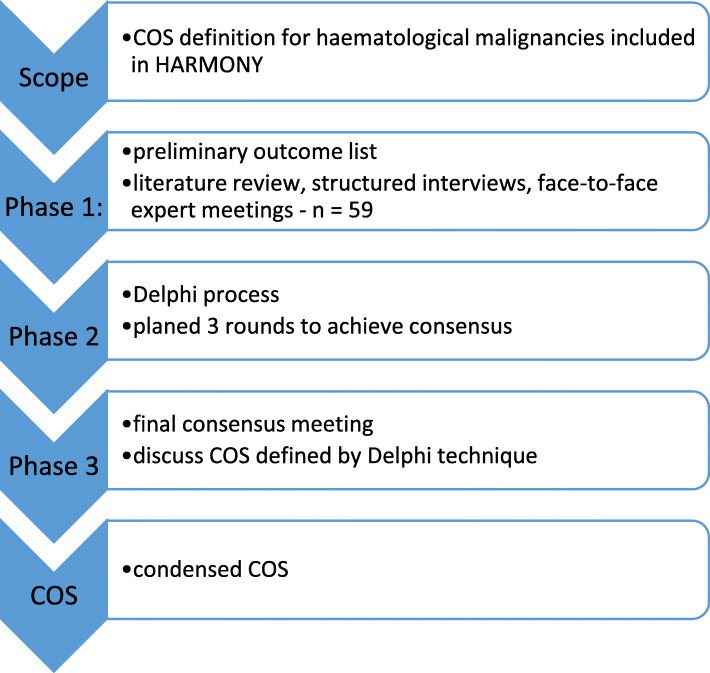
Steps of the planned Delphi process

COS development will follow recommendations of the Core Outcome Measures in Effectiveness Trials (COMET) initiative from the international Core Outcome Set Standards for Development (COS-STAD) [3, 4]. The Delphi method will be used to achieve a consensus from different stakeholder groups. A prospective study protocol was registered in the COMET database (http://www.comet-initiative.org/studies/details/1347). The protocol was written in accordance with the Core Outcome Set-Standardised Protocol (COS-STAP) recommendations [5] in cooperation between WP 2 and WP 6. In Fig. 1, each step of the study is illustrated.

### Study design

Recruitment of participants mainly takes place from members of the HARMONY WPs, but participants outside the HARMONY Alliance are also invited to take part in the Delphi survey within their stakeholder group. At least three rounds of the Delphi survey are planned to achieve consensus. At the end, a final face-to-face consensus meeting will take place.

### Scope of the COS

The COS-identified AML will be crucial not only for the HARMONY project but for all future analyses. As the definition of COS will determine what data are captured for AML, it will also impact how the analyses will be available in the future. Consequently, COS will directly impact future data sets available to HARMONY and also the research community. Additionally, future work conducted by HARMONY partners and other stakeholders in the AML field will be directly impacted by the defined COS.

### Participants

1. Patients
  Every patient older than 18 years with AML can participate. Different subtypes of AML are equally included, regardless of previous treatments, including stem cell transplantation. Patients treated as outpatients were included as well as patients treated in hospitals. Owing to the use of English for the Delphi survey, participation is limited to patients understanding English.
2. Clinicians and clinical researchers
  Every clinician within or outside the HARMONY Alliance who has experience in AML treatment can participate.
3. Drug developers
  Members of European Federation of Pharmaceutical Industries and Associations (EFPIA) and part of the HARMONY Alliance are invited to participate as well.
4. Regulators

Recruitment of participants will be performed within the HARMONY Alliance with the support of WP 7, which is responsible for dissemination, communication and training within the HARMONY Alliance. WP 6 provides assistance in promotion for the survey.

To recruit health-care professionals, AML key opinion leaders will be contacted and will be asked to invite other professionals within their peer groups to take part in the Delphi survey. Thus, participants outside the HARMONY Alliance are welcome to take part in the Delphi survey. Patient recruitment will be performed with the support of patient advocates and several patient organizations, like Acute Leukemia Advocates Network or LeukaNet. Especially in the recruitment of patients, social networks and internal communication tools will be used to spread the information and invitations for the Delphi survey. In the next rounds, personal mail reminders will be sent out to enhance the response rates.

### Trial registration

This project has been registered (May 2019) in the database of the COMET initiative

(http://www.comet-initiative.org/studies/details/1347).

### Study management group

As recommended by the COMET initiative, a study management group has been assembled to oversee the project [3]. The group comprises a study coordinator, a hematologist with leading roles in AML treatment and clinical trials, a drug developer with experience in past and current trials, patient advocates and methodological experts with experience of systematic reviews and Delphi studies. The role of the study management group is to support the development of the study protocol and to review the list of outcomes and the associated lay versions and descriptions.

### Selection of the preliminary outcome list for AML

So far, the empirical basis for identifying a long list of preliminary outcomes relevant in AML for the Delphi study has been threefold:

1. A literature research was conducted in the COMET database to get an overview of the cancer outcomes already used in existing clinical trials. Therefore, all relevant trials for outcome set definition in AML were collected to October 17, 2017 [6]. The primary AML outcome list was generated by extracting outcomes from the COMET database research and review of published literature [7–14].
2. Several semi-structured interviews of clinical public and private key opinion leaders were conducted to assess the initial selection of the outcome parameters, and additional outcomes were supplemented. This was followed by several face-to-face meetings to further expand and discuss the potential outcome list, including a multi-stakeholder group workshop and a further meeting with European AML key opinion leaders within the scope of the HARMONY Alliance.
3. Patient representatives and patients were consulted to include patients’ perspective. The preliminary outcome list was complemented by including additional outcomes and revising in accordance with patients’ comments.

On the basis of new scientific knowledge [15] and recommendations of the COMET initiative [3], the outcome list was reworked. Prognostic factors (e.g., age and gender) were removed from the list. Tools of “how to measure” an outcome (e.g., quality-of-life questionnaires) were also removed. Instead, AML concepts (outcomes related to patients’ perception of their symptoms, functioning and health-related quality of life) were included.

After the pre-Delphi stage, a list of 59 outcomes, each with a short plain language description, grouped into eight domain categories was completed. You can find the list used for this Delphi survey as Additional file 1.

### Delphi process

The Delphi survey will be managed online by using DelphiManager software maintained by the COMET initiative [6]. Invitations with a registration link will be sent out. After registration, every participant will receive a unique identification code to take part in the Delphi survey. For registration, participants are asked to fill in their email address, their stakeholder group and their home country. The webpage includes a description of aims and objectives of the survey and gives an explanation about how to complete the online Delphi survey. In every round, the participants will be asked to rate the importance of each outcome on the basis of their personal experiences. Each outcome will be ranked into three categories (“not important”, “important, but not critical” and “critical”) by using a 9-point Likert scale.

After the first round, a descriptive statistic for every stakeholder group will be provided. Only participants who completed the first round will be invited to take part in the second Delphi round. In the following rounds, participants will revise their answers by taking the previous results into account. The process is stopped after pre-defined consensus criteria.

### Consensus criteria

To reduce potential bias in interpretation of the results, a clear consensus definition is important. We will use three categories of consensus that were already used in previous works [16].

1. “Consensus in” means 70% or more of all respondents scored the outcome as critically important and 15% or fewer of all respondents scored the outcome as of limited importance.
2. “Consensus out” means 70% or more of all respondents scored the outcome as of limited importance and 15% or fewer of all respondents scored the outcome as critically important.
3. No consensus.

Outcomes that do not achieve consensus through several Delphi rounds will be discussed in a final face-to-face consensus meeting to finally ratify the AML COS. Representatives from all participating stakeholder groups will be part of this meeting.

### Analysis

Analysis of the Delphi study will use descriptive statistics. The results for each Delphi round, for each outcome and for each stakeholder group will be presented in frequency tables. The analysis of the Delphi survey will be performed by using the R statistical software version 3.5.2.

As an exploratory analysis, we additionally identify outcomes considered important for patients. The median Likert score for the patient group at the end of each round will be calculated, and those outcomes achieving a median of at least 7 will be considered important for patients and will be included in the COS. In this way, patient-important outcomes can be separately discussed in the final consensus meeting. Attrition bias will be investigated by comparing results across participants who complete successive rounds versus those who withdraw at round 2 or 3. Until now, no valuable data are available about the best group size for a Delphi survey; in this AML pilot Delphi, a group size of 20–50 participants should be achieved.

## Discussion

The described modified Delphi process will help to define a COS for AML on the basis of consensus of different stakeholder groups. To ensure the impact of patients’ involvement, an additional criterion in analysis will mark the outcomes with special interest for patients.

The language used in this Delphi survey is English. Therefore, participation is limited to men and women with a sufficient command of English to read and understand the survey. This constitutes a restriction of the study, but translation in other European languages is not proposed for the pilot Delphi.

A further limitation of the study is recruitment of participants in the course of the HARMONY communication platform and patient umbrella organizations.

Participants will be asked about their home country at registration.

The anticipated way of COS development ensures that clinicians, EFPIA members, health authorities and patients have an equal share in each stage of the process.

Carrying out a pilot Delphi will greatly inform the design and success of further Delphi surveys for COS definition for other hematological diseases considered in HARMONY.

The defined COS should be considered a minimum set of outcomes for AML, which will be collected and reported in future clinical trials, real-world evidence research and observational studies. In addition, further outcomes of special interest that are deemed relevant can be included in future research.

After the definition of a COS in AML, the next challenge will be the implementation of these outcomes in clinical guidelines and at last in clinical practice. Finally, patient treatment and patient satisfaction can be improved by reducing heterogeneity among clinical trials. The measured outcomes will be more easily comparable, meta-analyses of pooled studies will be more meaningful, and the level of evidence of future guidelines will be improved.

## Trial status

At the time of manuscript submission, the Delphi survey was open to recruitment. The starting date was April 12, 2019, and the approximate closing date was June 9, 2019. Protocol version 17 was published April 12, 2019.

## Supplementary information

**Additional file 1.** Preliminary acute myeloid leukemia (AML) outcome list.

**Additional file 2.** Core Outcome Set-Standardised Protocol (COS-STAP) checklist

## Data Availability

Not applicable.

## Abbreviations

AML: Acute myeloid leukemia
COMET: Core Outcome Measures in Effectiveness Trials
COS: Core outcome set
EFPIA: European Federation of Pharmaceutical Industries and Associations
HARMONY: Healthcare Alliance for Resourceful Medicine Offensive against Neoplasms in Hematology
WP: Work Package
