# Supplementary material for: Core outcome set measurement for future clinical trials in acute myeloid leukemia: the HARMONY study protocol using a multi-stakeholder consensus-based Delphi process and a final consensus meeting
Source: Trials. 2020 May 27;21:437. doi: 10.1186/s13063-020-04384-1 (PMC7251906; doi:10.1186/s13063-020-04384-1)
Supplement: Supplementary file 2 — Additional file 2. Core Outcome Set-Standardised Protocol (COS-STAP) checklist [file 13063_2020_4384_MOESM2_ESM.docx]

**Core Outcome Set-STAndardised Protocol Items: COS-STAP Statement**

|  |  |  |  |
| --- | --- | --- | --- |
| Title/Abstract |  |  | manuscript page |
| Title | 1a | Identify in the title that the paper describes the protocol for the planned development of a COS | 1 |
| Abstract | 1b | Provide a structured abstract | 1-3 |
| Introduction |  |  |  |
| Background and objectives | 2a | Describe the background and explain the rationale for developing the COS, and identify the reasons why a COS is needed and potential barriers to its implementation | 4-6 |
|  | 2b | Describe the specific objectives with reference to developing a COS | 4-6 |
| Scope | 3a | Describe the health condition(s) and population(s) that will be covered by the COS | 5 |
|  | 3b | Describe the intervention(s) that will be covered by the COS |  |
|  | 3c | Describe the context of use for which the COS is to be applied | 5 |
| Methods |  |  |  |
| Stakeholders | 4 | Describe the stakeholder groups to be involved in the COS development process, the nature of and rationale for their involvement and also how the individuals will be identified; this should cover involvement both as members of research team and as participants in the study | 6-7 |
| Information source | 5a | Describe the information source that will be used to identify the list of outcomes. Outline the methods or reference other protocols/papers | 8-9 |
|  | 5b | Describe how outcomes may be dropped/combined, with reasons | 8-9 |
| Consensus process | 6 | Describe the plans for how the consensus process will be undertaken | 9-10 |
| Consenus definition | 7a | Describe the consensus definition | 10 |
|  | 7b | Describe the procedure for determining how outcomes will be added/combined/dropped from consideration during the consensus process | 10-11 |
| Analysis |  |  |  |
| Outcome scoring/feedback | 8 | Describe how outcomes will be scored and summarised, describe how participants will receive feedback during the consensus process | 10-11 |
| Missing data | 9 | Describe how missing data will be handled during the consensus process |  |
| Ethics and Disseminations |  |  |  |
| Ethics approval/informed consent | 10 | Describe any plans for obtaining research ethics committee/institutional review board approval in relation to the consensus process and describe how informed consent will be obtained (if relevant) | n.a. |
| Dissemination | 11 | Describe any plans to communicate the results to study participants and COS users, inclusive of methods and timing of dissemination | 11 |
| Administrative information |  |  |  |
| Funders | 12 | Describe sources of funding, role of funders | 13 |
| Conflict of interests | 13 | Describe any potential conflicts of interest within the study team and how they will be managed | 13 |
